# Accuracy of budget impact estimations and impact on patient access: a hepatitis C case study

**DOI:** 10.1007/s10198-019-01048-z

**Published:** 2019-04-05

**Authors:** Joost W. Geenen, Cornelis Boersma, Olaf H. Klungel, Anke M. Hövels

**Affiliations:** 1 Division of Pharmacoepidemiology and Clinical Pharmacology, Utrecht Institute for Pharmaceutical Sciences (UIPS), Utrecht University, Universiteitsweg 99, 3584CG Utrecht, The Netherlands; 2 Division of Global Health, Department of Health Sciences, University of Groningen, University Medical Center Groningen, Antonius Deusinglaan 1, 9713 AV Groningen, The Netherlands; 3 Health-Ecore, 1e Hogeweg 196, 3701 HL Zeist, The Netherlands

**Keywords:** Hepatitis C, Budget impact, Budget impact accuracy, Direct-acting antivirals, Affordability, Pharmaceuticals, I180, H51

## Abstract

**Background:**

High budget impact (BI) estimates of new drugs limit access to patients due to concerns regarding affordability and displacement effects. The accuracy and methodological quality of BI analyses are often low, potentially mis-informing reimbursement decision making. Using hepatitis C as a case study, we aim to quantify the accuracy of the BI predictions used in Dutch reimbursement decision-making and to characterize the influence of market-dynamics on actual BI.

**Methods:**

We selected hepatitis C direct-acting antivirals (DAAs) that were introduced in the Netherlands between January 2014 and March 2018. Dutch National Health Care Institute (ZIN) BI estimates were derived from the reimbursement dossiers. Actual Dutch BI data were provided by FarmInform. BI prediction accuracy was assessed by comparing the ZIN BI estimates with the actual BI data.

**Results:**

Actual BI, from 1 Jan 2014 to 1 March 2018, was €248 million whilst the BI estimates ranged from €388–€510 million. The latter figure represents the estimated BI for the reimbursement scenario that was adopted, implying a €275 million overestimation. Absent incorporation of timing of regulatory decisions and inadequate correction for the introduction of new products were main drivers of BI overestimation, as well as uncertainty regarding the patient population size and the impact of the final reimbursement decision.

**Discussion:**

BI in reimbursement dossiers largely overestimated actual BI of hepatitis C DAAs. When BI analysis is performed according to existing guidelines, the resulting more accurate BI estimates may lead to better informed reimbursement decisions.

**Electronic supplementary material:**

The online version of this article (10.1007/s10198-019-01048-z) contains supplementary material, which is available to authorized users.

## Introduction

The role of budget impact (BI) in healthcare decision-making varies across different jurisdictions as recent reviews indicate [1–4]. Germany and the USA are examples of jurisdictions that do not have a formal or informal role for budget impact in decision-making. Other countries, for example The Netherlands, France and Australia, do have guidance or even legislation on BI, but the actual role of BI or the impact on decision-making remains rather informal and, moreover, politically driven [1–4]. On the other end of the spectrum, England has one of the best defined systems with a clear role for BI in healthcare decision making [2, 3]. In general, however, there is an informal role for BI and its contribution to reimbursement decisions often remains unclear. As a result of that, the role of BI in decision-making remains an important topic for debate [1, 5, 6]. In particular, the growing attention for healthcare and pharmaceutical expenditures in combination with price negotiation mechanisms increasingly raises questions about the role of cost-effectiveness (CE).

Whilst the role of BI is often unclear in reimbursement decision-making, there are ample examples where BI did play a significant role in either the reimbursement decisions or where high BI estimates resulted in restricted reimbursement for a specific patient population [6–11]. Recently, the introduction of new, very effective but high priced Direct-acting antivirals (DAA) in hepatitis C sparked worldwide affordability concerns and access restrictions [7, 8]. Also in oncology, patients have limited access to many high priced products due to concerns regarding affordability as a result of high BI [9–11].

Especially in the hepatitis C case, the cost-effectiveness of the innovations were generally regarded as positive and medical need was high [12–16]. The future will bring new products with potentially high short-term BI, which could spark further BI-guided restrictions and will call for further deliberation of the role of affordability in the political and societal debate and as such of relevant meaning in healthcare decision-making [17, 18]. Therefore, clarity on the role and hierarchy of CE vs BI will not only be of interest but seems to become very important in informing reimbursement decisions.

Unfortunately, the (methodological) quality and accuracy of BI analysis does not seem to match the proven scientific rigor of CEAs [6, 19, 20]. A review by Van de Vooren et al. [21] reports that (methodological) quality of many published BI analyses is poor. Furthermore, Broder et al. and Cha et al. illustrate that the accuracy of BI predictions is regarded as low [22, 23]. These observations, in light of the increased debate on drug prices, growing interest in price negotiation, BI of pharmaceuticals as part of healthcare budgets (e.g. Hospital) and, therefore, burden to societies, warrant questions about whether BI is being used properly and what the extent of influence is on patient access.

In this paper, the accuracy and role of BI in reimbursement decisions is investigated by assessing the life cycle of hepatitis C DAAs in the Netherlands. This case was selected as there were concerns for an extremely high BI (up to €1.78 billion). The final reimbursement decision of Sofosbuvir (Sovaldi), the first DAA, resulted in restricting treatment to the most critically ill whilst this seems rather irrational from a cost-effectiveness perspective and was likely triggered by other elements such as price, BI considerations and affordability discussions [12, 13, 24–28].

The aim of this hepatitis C case study is twofold: First, we aim to quantify the accuracy of the BI predictions used for informing the Dutch reimbursement decisions. Second, we attempt to characterize the influence of market-dynamics on actual BI and the way these are implemented in the BI predictions. This includes, for example, timing of regulatory decisions, influence of introductions of new hepatitis C products and the influence of a restricted reimbursement decision that limits the product’s indication.

## Methods

### Product inclusion

We included hepatitis C DAAs that were mainly designated a standalone option for treatment of hepatitis C according to the EASL guidelines, thereby not considering co-treatment with ribavirin and/or pegylated interferon [29–32]. We subsequently excluded products that were not introduced or not used in the Netherlands in the period from 1 Jan 2014 to 1 March 2018. Lack of use or introduction was based on a publicly available national drug information system (GIP), which has national coverage and is maintained by the National Health Care Institute (ZIN) [33].

Daclatasvir (Daklinza) and simeprevir (Olysio) were excluded as these products are mainly used in combination with sofosbuvir (Sovaldi) but not as monotherapy. The sofosbuvir/velpatasvir/voxilaprevir (Vosevi) combination was not introduced and is thus excluded. Sovaldi, sofosbuvir/ledipasvir (Harvoni), ombitasvir/paritaprevir/ritonavir (Viekirax) + dasabuvir (Exviera), sofosbuvir/velpatasvir (Epclusa), elbasvir/grazoprevir (Zepatier) and glecaprevir/pibrentasvir (Maviret) were included.

### BI data and BI estimation accuracy

The actual Dutch BI data was provided by FarmInform [34]. The population-level data of FarmInform comprises of monthly volume of all prescription drugs in the in- and outpatient setting multiplied by the respective monthly list price in the Netherlands [35]. Validity of the data is ensured as the data is crosschecked with patient-level data that is representative of the Netherlands (PHARMO) [36, 37]. As DAAs target specific hepatitis C viral proteins, off-label use of DAAs is highly unlikely and we, therefore, assume that all DAA BI is used for treatment of hepatitis C.

The BI estimates used to inform the reimbursement decisions of hepatitis C therapy in the Netherlands were collected from the published and publicly available ZIN reimbursement dossiers [12, 13, 24–27, 38]. These dossiers typically project the BI for the 3 years after publication of the dossier. The ZIN BI estimation format and methodology are based on the most recent ISPOR guidelines for conducting BI analysis [39, 40]. BI is based on market potential: it accounts for expected patient populations and one or more treatment regimens and associated costs [39, 40]. Correction should be performed for 1st in class vs subsequent introductions by making assumptions regarding the market penetration [39]. It is also recommended to include the effects of restrictions in indication due to the eventual reimbursement decision [39].

The treatment regimens or subpopulations that are mentioned in the reimbursement dossiers are based on (combinations of) METAVIR score, genotype, IFN or ribavirin co-medication and prior treatment experience. From the dossiers, estimated BI, population size and average treatment costs were recorded, as well as the subpopulations and the aforementioned characteristics these estimations were based on. The BI prediction accuracy was then assessed by comparing the ZIN BI estimates with the real world actual BI data for all included products.

### Treatment indication and resulting access

As there was a potential for significant budget impact, the Sovaldi reimbursement decision stated treatment was to be restricted to more severely ill patients [28]. To investigate the effect and extent of this reimbursement restriction and the development of access when DAAs without restrictions were introduced, we aimed to quantify the amount of DAA access by translating actual BI to a number of patients treated.

Number of patients treated was calculated as follows:

$${\text{Number of patients treated}} = \frac{\text{Budget impact}}{\text{Average treatment cost per patient}}.$$

As BI is known from the actual BI data, average treatment cost per patient had to be established. Each product has a standard treatment duration (12 weeks for most products, 8 weeks for Maviret) that can be multiplied by the known list price to obtain the cost of treating one patient. Some subpopulations, however, require a longer treatment duration:

- Genotype: The hepatis C virus is classified in six genotypes. They differ in susceptibility to (DAA) treatment as GT 3 typically requires longer treatment [29, 31, 32, 41, 42].
- Severity of disease: More severe disease evidently warrants not only (more) immediate treatment but also longer treatment [29, 31, 32].
- Prior treatment: Treatment experienced patients in some cases require longer treatment [31, 32].

Chronic hepatitis C disease severity is frequently categorized using the well-validated METAVIR scoring system [43, 44]. This five point scale distinguishes between various stages of liver fibrosis where F0 = no fibrosis, F1 = portal fibrosis without septa, F2 = portal fibrosis with rare septa, F3 = numerous septa without cirrhosis, F4 = cirrhosis [43, 44]. For clarity, we do not consider extrahepatic complications of hepatitis C, and thus solely reflect disease severity by means of METAVIR score.

EASL guidelines on treatment of particular METAVIR scores changed particularly:

- EASL 2014 and 2015: All patients with chronic liver disease related to HCV should be considered for therapy. Treatment should be prioritized in patients with METAVIR score F3 and F4. Treatment is justified in patients with METAVIR score F2. The timing and nature of therapy for patients with METAVIR score F0 + F1 debatable, and informed deferral can be considered [29, 30].
- EASL 2016: All patients with chronic liver disease related to HCV must be considered for therapy. Treatment must be considered without delay in patients with METAVIR score F2–F4 [31].
- EASL 2018: All patients with HCV infection should be treated. Treatment must be considered without delay in patients with METAVIR score F2–F4 [32].

The influence of these factors on treatment duration changed over time as the leading European Association for the study of the Liver (EASL) hepatitis C guidelines changed and new DAAs were introduced [29–32]. Supplemental Table 1 summarizes the major exceptions regarding treatment duration for various subpopulations.

### Mean treatment costs per product

As, in for example the case of Harvoni, METAVIR stage F4 indicates a longer treatment duration, a larger proportion of patients with stage F4 would increase average treatment costs. This is of particular interest as the Harvoni and Viekirax + Exviera, dossiers specifically address 3 national BI scenarios based on only treating patients with F4 + F3 (scenario A), F4–F2 (scenario B) and F4–F0 (scenario C) [12, 26]. Average treatment costs thus differ per scenario. To be able to adjust average treatment costs to different scenarios, the ZIN BI calculations had to be recreated so that the influence of different populations could be assessed. Average treatment costs per patient were solely recreated using the assumptions and data from the respective reimbursement dossiers.

In the reimbursement dossiers, the following assumptions for the Dutch setting were made: The genotype distribution is 49% GT1, 10% GT2, 29% GT3 and 11% GT4, GT5 and GT6 are very rare in the Netherlands [13, 41, 45]. Sovaldi treatment regimens for GT2 and GT3 are IFN free whilst for GT1, GT4–6 30% of patient will be treated with an IFN free regimen [13]. The METAVIR distribution is assumed to be 24.9% F0, 26% F1, 16.1% F2, 16.8% F3, 16.2% F4 [12]. ZIN states that the total number of chronic HCV patients, thus from F0–F4 and GT1–4, in The Netherlands is between 2000 and 3000 (29). With a population of 16.9 million at the time of publication, this implies a prevalence of 0.012–0.018% [46].

The reimbursement dossiers of Sovaldi, Harvoni and Viekirax + Exviera provided detailed insights into the types of patients receiving specific treatment durations, costs and various calculations [12, 13, 26]. For these products we were, therefore, able to calculate scenario/population dependent average treatment costs.

For Epclusa, Zepatier and Maviret, only a short reimbursement report with rudimentary budget impact prediction was published [24, 25, 27]. These BI estimations lacked detailed assessments of treatment duration per subpopulation. In our analysis, we, therefore, assumed the following:

- Maviret: We assume that all patients are treatment naïve as we have no valid data regarding the distribution of treatment experienced vs treatment naïve patients. That implies that, according to the ZIN reimbursement dossier, all F0–F3 patients receive 8 weeks of treatment and F4 patients are treated for 12 weeks [25].
- Zepatier: The only exceptions to the standard 12 weeks treatment are in cases where HCV RNA > 800,000 IU/ML [27, 31]. As we have no clear data on the number of patients in the Dutch setting, we disregard this exception and assume all patients are treated for 12 weeks.
- Epclusa: There are no exceptions to the standard 12 weeks treatment duration so we assume that all patients are treated for 12 weeks [24, 31].

For our base–case analysis, we take the average of the estimated patient population size at 2500 (range 2000–3000). We, furthermore, use treatment costs of the F4–F2 (B) scenario. Changes in list-price over time were corrected using the G-standard, a database that contains the monthly list-prices of all Dutch prescription drugs so that the correct amount of patients are calculated [35]. We did not incorporate EASL guideline changes in our analyses.

### Sensitivity analyses

By means of sensitivity analysis, we investigate the scenarios proposed in the reimbursement dossier that we did not use as base–case scenario. We thus investigate the influence of a different population size and treatment cost. For population size, we take the minimum (2000) and maximum (3000) values of the range that was estimated in the reimbursement dossiers. Furthermore, we investigate the influence of average treatment cost per patient by using the F4 + F3 (A) and F4–F0 (C) scenarios. Additionally, we perform an analysis with the absolute minimum treatment cost where we assume that no patients get extended treatment regimens for any of the included products. Finally, we assess the influence of GT3 prevalence on average treatment costs as this genotype generally warrants a longer, and thus a more costly treatment. We, therefore, increase GT3 prevalence from 30 to 50%, which is higher than the reported GT3 prevalence in any European country, decrease GT1 prevalence from 50 to 30% and recalculate the average treatment costs [42].

## Results

### Accuracy of BI estimates

**Fig. 1:**
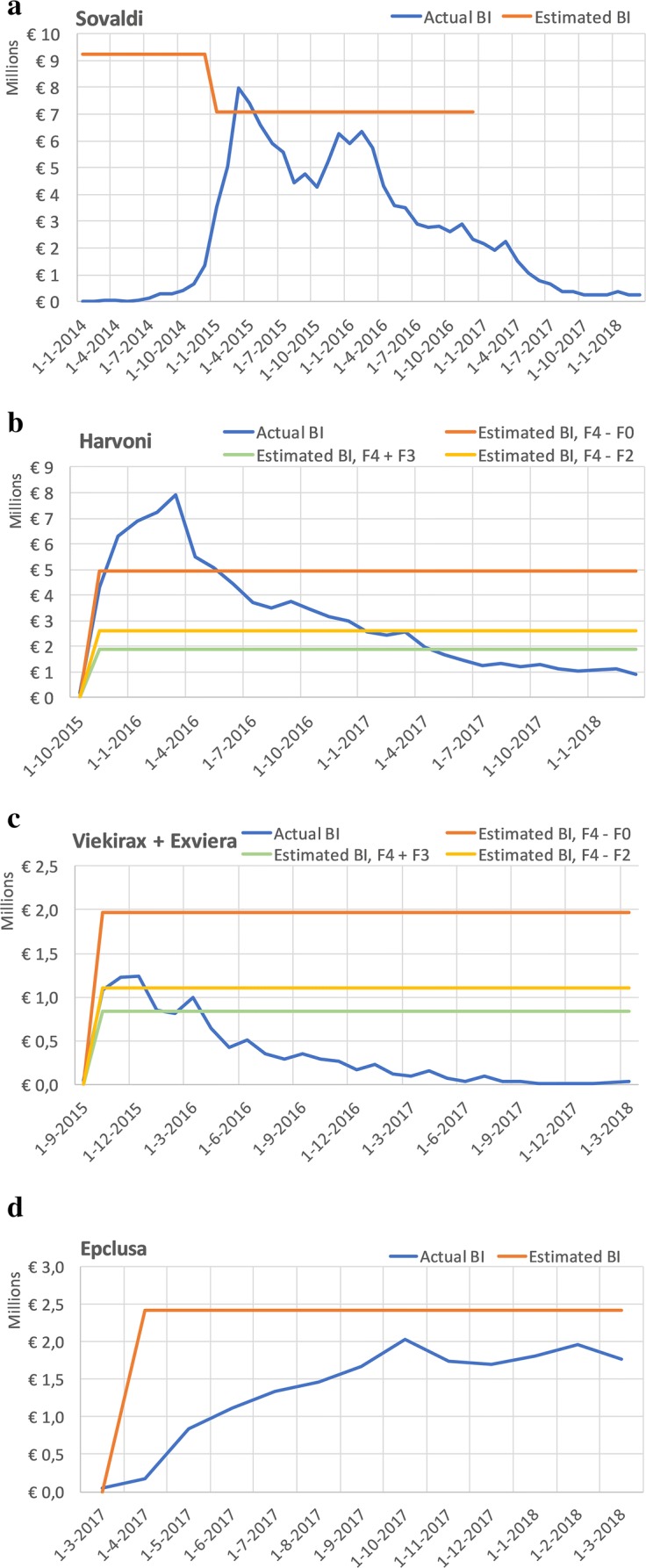
Estimated BI vs Actual BI, values are in € millions and per month

We compared the estimated BI from reimbursement reports with the actual BI. The estimated BI timeline starts at the date at which national reimbursement was granted except when an explicit period was mentioned. As mentioned before, the Sovaldi reimbursement decision restricted treatment to F4 + F3. The eventual reimbursement decisions for Harvoni and Viekirax + Exviera were without restrictions, meaning that scenario C (F4–F0) had been adopted. Maviret, Epclusa and Zepatier were also reimbursed without restriction but for these products, no a priori scenarios were made. Figure 1 displays the actual BI and estimated BI for the only four products with a reported estimated BI. BI overestimation is apparent for all products with respect to their eventual reimbursement decision. For Harvoni and Viekirax + Exviera, the lower F4 + F3 scenario is closer to the actual BI than the adopted scenario.

**Fig. 2:**
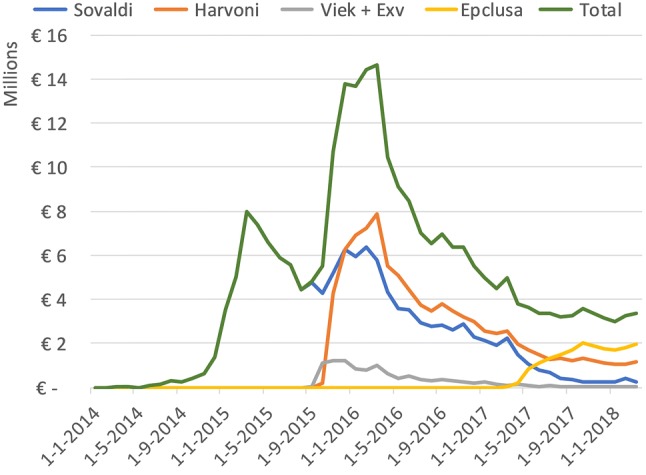
Total monthly BI. Total BI and Sovaldi BI overlap until 1 Sep 2015 as Sovaldi is then the only product

**Fig. 3:**
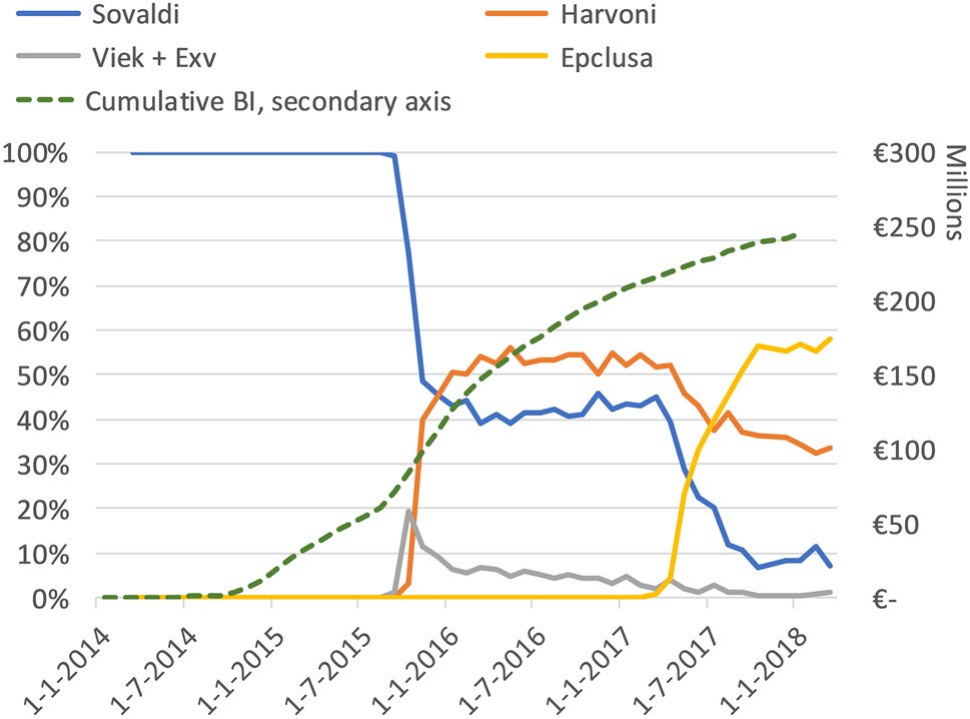
Share of BI per product on the left vertical axis. Cumulative BI (in millions) of all four products is shown in the right vertical axis

Figure 2 combines the monthly BI of the individual products and shows the total BI of these four products. BI initially peaks with the introduction of Sovaldi to a monthly BI of about €8 million in the first quarter of 2015. Then, with the introduction of Viekirax + Exviera and Harvoni, a monthly BI of €14 million is reached and sustained for 4 months. In Fig. 3 we display the relative market share of the four products with an estimated BI, including the cumulative BI. The cumulative BI shows that in about 4 years, €250 million was spent on the four DAAs. The assumed market share of Harvoni (35%) and Viekirax + Exviera (35%) was, in reality, between 40–60% and < 10%, respectively.

The total actual BI, estimated BI and total absolute deviation per individual product and for the total cohort are denoted in Table 1. Table 2 displays the standard treatment duration per product, the eventual average treatment costs per product and, if applicable, per scenario. Even with the most modest treatment scenario (F4 + F3), treatment costs were overestimated at €153 million. When extending to the adopted and most inclusive treatment regimen (F4–F0), total overestimation increases to €275 million. As the time between introduction of Sovaldi (1 Jan 2014) until the last data-point (1 Mar 2018) is slightly over 4 years, the annual overestimation of hepatitis C treatment costs are around €38–€69 million.

**Table 1:** Overview of Actual BI, Estimated BI and the difference between actual- and estimated BI. A negative difference implies an overestimation of BI

Product (METAVIR score)	Actual BI (€)	Estimated BI (€)	Difference (€)
Sovaldi	128,692,991	281,166,336	− 165,151,871
Harvoni (F4 + F3)	91,461,345	54,495,833	36,796,849
Harvoni (F4–F2)	91,461,345	76,004,167	15,457,178
Harvoni (F4–F0)^a^	91,461,345	143,550,000	− 52,257,318
Viekirax + Exviera (F4 + F3)	10,557,104	24,166,667	− 13,609,563
Viekirax + Exviera (F4–F2)	10,557,104	32,020,833	− 21,463,730
Viekirax + Exviera (F4–F0)^a^	10,557,104	57,033,333	− 46,476,230
Epclusa	17,632,950	29,000,000	− 11,413,049
Total (F4–F3)	248,344,389	388,828,836	− 153,377,635
Total (F4–F2)	248,344,389	418,191,336	− 182,571,472
Total (F4–F0)	248,344,389	510,749,669	− 275,298,468

^a^The eventual reimbursement decision

**Table 2:** Average treatment cost per patient per reimbursement scenario

Product	Treatment costs (F3 + F4) (€)	Treatment costs (F2–F4) (€)	Treatment costs (F0–F4) (€)	Standard treatment duration costs	Standard treatment duration (weeks)
Sovaldi	73,153	73,153	73,153	48,000	12
Harvoni	85,936	80,383	74,589^a^	51,750	12
Viekirax + Exviera	57,959	51,444	45,172^a^	39,400	12
Epclusa	45,999	45,999	45,999	45,999	12
Zepatier	41,397	41,397	41,397	41,397	12
Maviret	30,666	30,666	30,666	30,666	8

^a^The eventual reimbursement decision

### Analysis of market dynamics

**Fig. 4:**
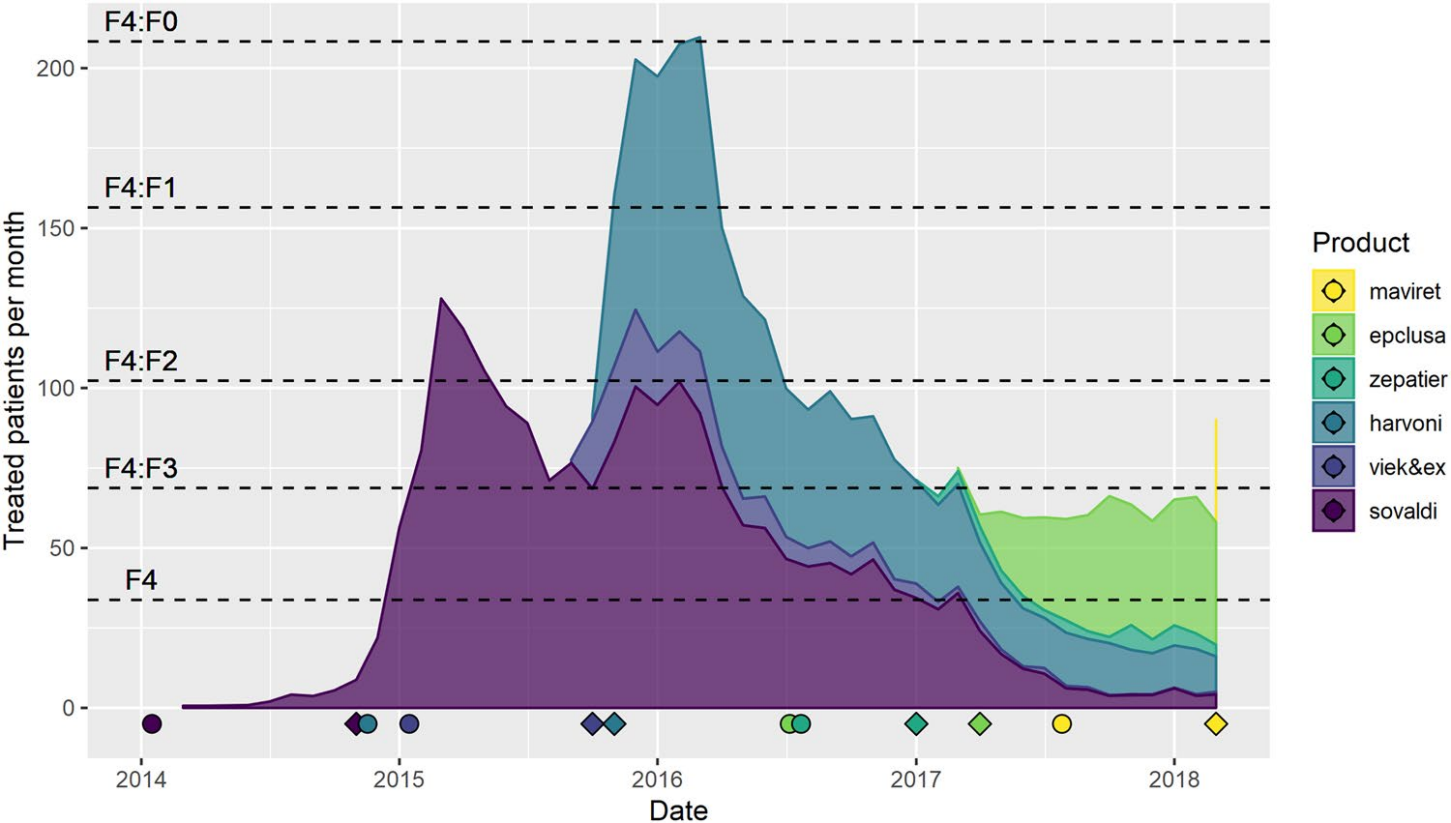
Treated patients over time, with monthly data. Dotted lines indicate the assumed number of patients with a specific METAVIR score. Circles indicate the date of EMA Marketing Authorization, diamonds indicate the date of positive reimbursement decision

The actual number of patients treated per month is visualized in Fig. 4. The theoretical number of patients in different METAVIR categories indicate the extent of treatment availability to various degrees of disease severity where we assume that treatment is prioritized according to METAVIR score. On the *x*-axis, date of granting European Medicines Agency (EMA) Marketing Authorization (MA) and the date of the formal initiation of national reimbursement are displayed. Note that a formal reimbursement status decision comes from the Minister of Health following an advice from ZIN.

It is evident that Sovaldi and Harvoni, at least until 2017, were most frequently used. Interestingly, for almost entire 2014, access was very limited due to absent reimbursement whilst EMA MA was granted in January. It seems that at least patients with METAVIR F4 and F3 were treated from 2015 onwards. For 2015 and 2016, treatment appears to have been extended to F2. The increase to F0 at the start of (unrestricted) reimbursement of Harvoni could be explained by the fact that treatment then became available for F2–F0 patients.

Apart from the initial peak of Harvoni and Sovaldi, broadening of the treatment population over time, as is recommended by the EASL guidelines and as is permitted by the reimbursement decisions of all products but Sovaldi, seems to be absent. This is apparent as from 2017 onwards, treated patient numbers remain stable at a level only encompassing the F3 and F4 patients. The rise in treated patients around the reimbursement date clearly confirm that in The Netherlands, access is governed by national coverage decisions and not by EMA MA.

We compared our patient estimates with the publicly available national GIP drug information system to ensure validity of our approach [33]. In supplemental Table 2 we extracted the annual number of users per product and they are reasonably comparable with our monthly estimates as displayed in Fig. 4.

### Sensitivity analyses

The different treatment costs used are displayed in Table 2. The sensitivity analyses show that the estimated size of the patient population has some influence with larger total populations yielding less access for more favorable METAVIR scores as a smaller fraction of the population appears to be treated. Supplemental Fig. 6 (treatment scenario C and base–case population size) shows the data based on the eventual reimbursement decisions for Harvoni and Viekirax & Exviera. Treatment scenarios A and C vary little compared to scenario B that was used as base–case. The minimum treatment cost scenario disregards any possibility for extended treatment durations for various subpopulations. This scenario thus results in a higher number of patients treated as treatment costs per patient were lower.

We explored the influence of GT3 prevalence on average treatment costs. For Harvoni and the various scenarios, average treatment costs increased with 7.5–13% whereas average Sovaldi treatment costs increased with 12–15%. Viekirax and Exviera are not recommended for GT3 and the reported treatment costs of Epclusa, Zepatier and Maviret are not influenced by genotype. Given that the GT3 prevalence increase from 30 to 50% is a 67% increase, we can conclude that average treatment costs are relatively insensitive to GT3 prevalence.

## Discussion

In a Dutch setting, we showed that BI estimates reported in ZIN reimbursement dossiers largely overestimated the actual BI for hepatitis C DAAs. Although the most severely ill patients did get access to the innovative hepatitis C therapies, access was initially not granted to the extent of the recommendations in the then prevailing EASL guidelines.

In the EU, the crude hepatitis C incidence is estimated to be 7.4 per 100,000 persons but with a very large spread (0.1–73.3) between countries, at least partly driven by varying quality of surveillance systems and data completeness [47–49]. ZIN dossiers as well as studies by Iyengar et al., Cornberg et al. and Saraswat et al. report larger potential eligible populations (22,000–28,000, 0.14–0.17%) than those that ZIN actually used for the BI calculations (2000–3000, 0.012–0.018%) [12, 48–50]. The former population, all those with chronic hepatitis C, should be treated according to the most recent EASL guideline. Interestingly, the estimates of 28,000 stated in the reimbursement dossiers are denoted as a scenario where the ‘indication is broadened’ to all hepatitis C patients. No further notion is given as to whether the presumable non-symptomatic patients are actually F0 patients. We can safely conclude that (1) current patient volumes have not been near this level and (2) the estimates of 2000–3000 are likely to be a conservative estimate.

In 2015, when Sovaldi was reimbursed, the BI and estimates of number of patients appear to be rather accurate as we see that, according to the reimbursed indication, treated patients are within the F4–F3/F2 range. Then, with the unrestricted reimbursement of Harvoni and Viekirax + Exviera, treated patients plateau to the predicted F4–F0 population for approximately 4 months. These observations would suggest that the ZIN estimate of 2000–3000 patients per year is quite accurate. From June 2016 onwards, however, patient numbers decline to an F4 + F3 level that is below the expected F4–F0 range. If we, for now, assume that the estimate of number of patients was indeed reasonably accurate, other factors must have been responsible for the large deviations between estimated and actual BI.

A first reason for this deviation could be the inadequate implementation of timing of regulatory decisions. The Sovaldi reimbursement dossier was published on 20 May 2014 based on which ZIN formally advised the minister of health on 23 May 2014. The final reimbursement status was granted per 1 Nov 2014. The delay between advice and reimbursement could have been unforeseen. The manufacturer and/or ZIN could, however, have assumed that reimbursement of Sovaldi during entire 2014 (MA was 16 Jan 2014) was highly unlikely. Still, the BI estimate assumed access during the entire year. This alone contributed to a €46 million overestimation which could have been prevented. Of course, the relevancy of this overestimation can be questioned as it is common-practice to start the period of estimation from the initiation of reimbursement. Applying this logic would shift the Sovaldi ‘Estimated BI’ line in Fig. 1 to the right and would cause a nearly equal overestimation from 01 Jan 2017 onwards due to declined market share.

In line with the overestimation due to a declined market share, inadequate correction for the introduction of new products could be a second reason. The Sovaldi BI estimations did not at all account for the introduction of new products in the same class whilst this was nearly inevitable as various manufacturers were in advanced stages of clinical development or regulatory approval [30, 51, 52]. The ISPOR BI guideline, to which ZIN refers in their own guidance on BI, states that an attempt should be made to forecast introduction of new interventions for the chosen time horizon [39]. Harvoni and Viekirax + Exviera were introduced nearly simultaneously and both were estimated to reach a market share of 35%. As our results show, the latter product only reached a small fraction of the estimated 35% whilst the former outperformed the expectations.

Of course, the patient estimates or the distribution over METAVIR scores could have been inaccurate. In addition, as DAA BI was in general overestimated and patient estimates are on the low side of estimations, we should consider the possibility that other forms of access restrictions were present. Stringent reimbursement criteria or volume caps issued to hospitals by payers might have been a factor in this regard as payers in The Netherlands have gained influence [53].

Transitioning towards the role BI played in governing access for this specific case study, we like to reiterate that actual BI stayed well below the BI estimations ZIN deemed realistic. Consequently, the actual BI was also considerably lower than alternative scenarios postulated by ZIN where a ‘broader DAA indication’ would be adopted. This, according to the reimbursement dossiers, could theoretically lead to a BI of €1.78 billion [12]. Such a wide call for treating all viraemic hepatitis C patients, culminating in the EASL’s 2018 guideline recommendation, has not been apparent in our data. Our data did, however, show that access is strictly governed by a positive reimbursement decision as a products’ BI is very low before it is reimbursed.

The reimbursement decision was, on average, taken 258 days after MA whilst the reimbursement dossier was published after on average 117 days. Price negotiations were conducted for all products for which the HTA report was used as guidance. There is, however, no report on the role that BI played in this process and whether BI estimations, which are known and proven to be uncertain, are necessary for either the reimbursement decision or the price negotiation. Additionally, one can extend this way of thinking with a debate on whether the 258 days of access restriction is worthwhile. This especially in light of the fact that DAAs are generally considered cost-effective [12–16].

The implications of over- and underestimations differ for various stakeholders. Patients and manufacturers of the specified products, would probably incur no real loss due to an initial underestimation. It could potentially even facilitate the reimbursement process whereas the contrary could be true for an initial overestimation. For payers, however, an underestimation could be more troublesome than an overestimation as the former could cause direct and measurable budgetary deficits. We have no evidence to support or quantify these statements, but it is known that payers and manufacturers have confidential price negotiations where their BI estimates together with other parameters serve some purpose for bargaining [54].

If there is a more aligned and agreed estimate of patient numbers, as the basis of a BIA, we believe that more accurate BI estimates are achievable. As is stated in the most recent BIA guideline, it is important to include treatment dynamics including new introductions and displacement effects as well as pricing dynamics to provide more accurate and meaningful BI estimates. This would allow for a more prominent role and value of BI in the decision-making process for reimbursement of different types of treatments (e.g. chronic and one-off).

Our study has various strengths. First, we used real world data to assess access using validated and monthly updated data. Our data covers in- and outpatient dispensing data, is irrespective of the healthcare provider or insurance company and, therefore, captures the entire Dutch DAA access data.

Second, we made an accurate representation of average treatment cost per patient using the distribution of several subpopulations. As our sensitivity analysis shows, not including patients with longer than typical treatment durations have a profound influence on outcomes.

Our study also has some limitations. First, several assumptions underlie population size estimates and the distribution of subgroup characteristics. We, therefore, crosschecked our estimates with the GIP-database which reports comparable figures [33]. We, furthermore, aimed to illustrate the influence of population size and treatment costs on outcomes by means of sensitivity analysis.

Second, our Dutch scenario induces limitations regarding generalizability. Of course, the Dutch healthcare and reimbursement system is not directly comparable to others but the, albeit imperfect, method of BI estimation is rather similar [21–23, 39].

Third, confidential discounts or rebates are not included in this analysis. In the Netherlands, the general outcome of price negotiations is published but the actual discount remains confidential. A study by Morgan et al. indicated that, in ten high-income countries (including The Netherlands), discounts are common and confidential discounts are most frequently in the 20–29% range but can also be substantially higher (> 60%) [55]. We thus know that the actual BI, as costs to society, are lower than presented here. Yet, we based our analyses on BI estimations that disregard potential discounts and rebates. Lack of inclusion of pricing agreements is, therefore, not a concern.

## Conclusion

The BI estimates published by ZIN provide a substantial overestimation of the actual BI with a deviation of between €153–€275 million. The number of treated patients remains low, especially in light of the much higher incidence of viraemic hepatitis C and the most recent EASL guideline recommending treatment for this entire population. Underlying patient number that were used for BI estimates seem to be at least somewhat overestimated but are probably not the sole cause of BI overestimations. Differences could potentially be caused by inadequate correction for (timing of) regulatory decisions, reimbursement for a limited indication and insufficient incorporation of the introduction of new products. These market dynamics are, to varying extent, unanticipated but could and should at least partly be corrected for. When BIA is performed according to existing guidelines, the resulting more accurate BI estimates can lead to better informed reimbursement decisions. Currently, it is unclear how the BI estimates informed the reimbursement decision and if different decisions would have been if more accurate BI estimates been had available. In light of increasing debate on prices, the (uncertain) role of the reimbursement dossier in confidential price negotiations and an increasing pressure on healthcare budgets in general, it is important to further develop an approach to use BI as a more integrated part of healthcare decision-making processes.

## Electronic supplementary material

Below is the link to the electronic supplementary material.

Supplementary material 1 Base–case (treatment costs and population size of 2500) as reference (TIFF 311 kb)

Supplementary material 2 Base–case treatment costs, maximum population size of 3000 (TIFF 296 kb)

Supplementary material 3 Base–case treatment costs, minimum population size of 2000 (TIFF 313 kb)

Supplementary material 4 Minimum treatment costs, base–case population (TIFF 307 kb)

Supplementary material 5 Scenario A treatment costs, base–case population (TIFF 310 kb)

Supplementary material 6 Scenario C treatment costs, base–case population (TIFF 309 kb)

Supplementary material 7 (DOCX 14 kb)

Supplementary material 8 (DOCX 12 kb)
