# Supplementary figures and images for: Accuracy of budget impact estimations and impact on patient access: a hepatitis C case study
Source: Eur J Health Econ. 2019 Apr 5;20(6):857–67. doi: 10.1007/s10198-019-01048-z (PMC6652171; doi:10.1007/s10198-019-01048-z)

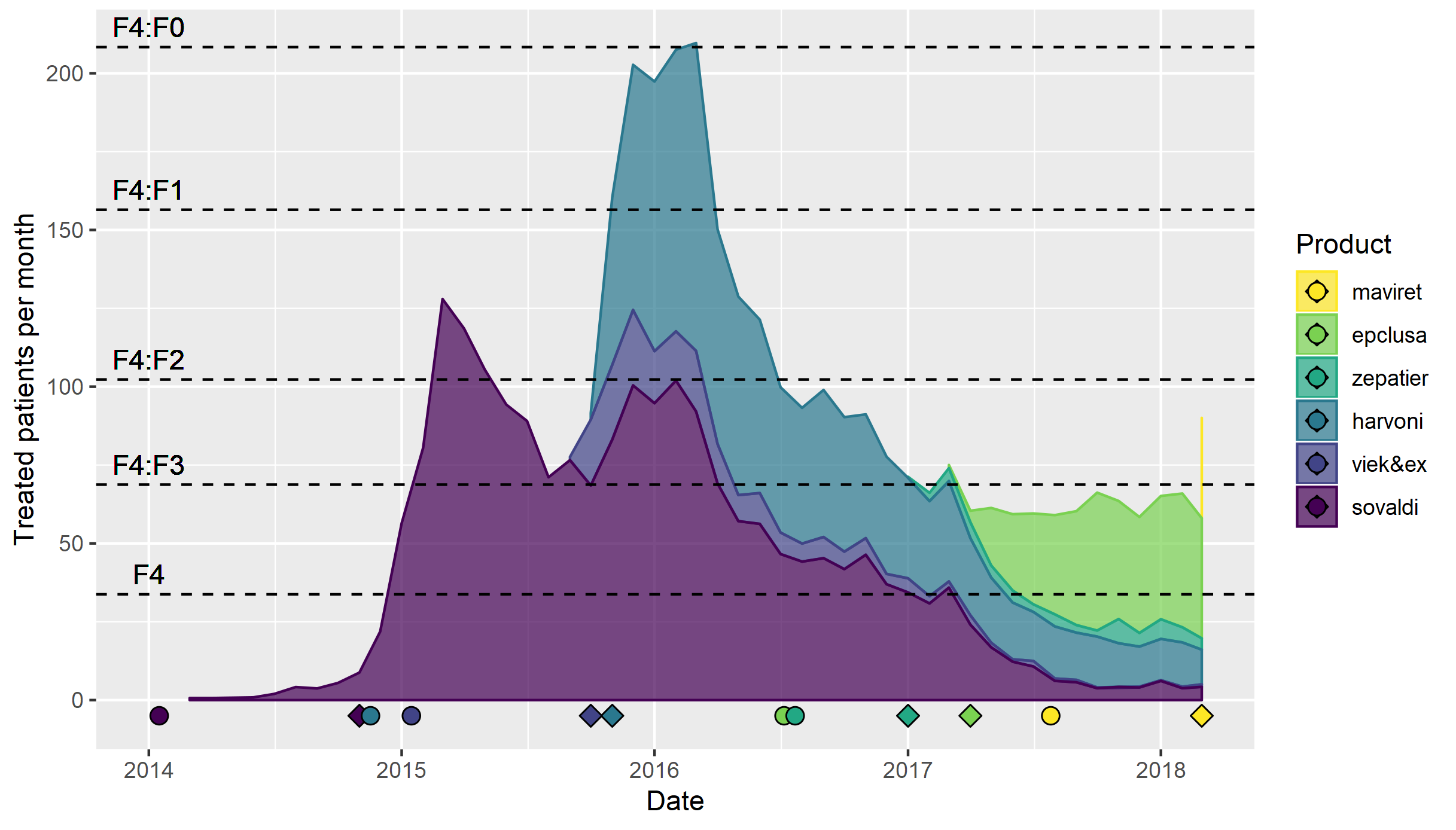

Supplement: Supplementary file 1 — Supplementary material 1 Base–case (treatment costs and population size of 2500) as reference (TIFF 311 kb) [file 10198_2019_1048_MOESM1_ESM.tif]

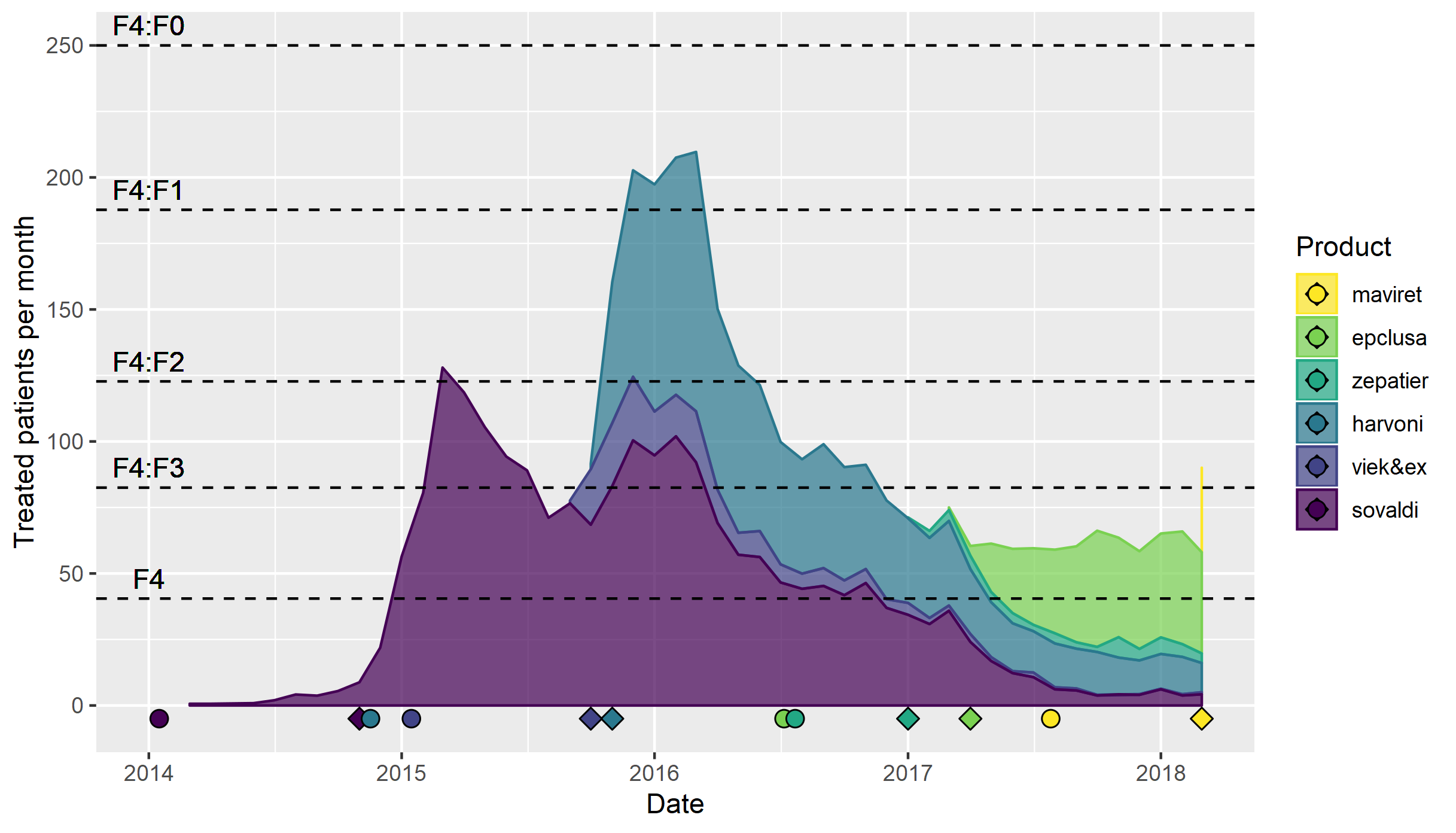

Supplement: Supplementary file 2 — Supplementary material 2 Base–case treatment costs, maximum population size of 3000 (TIFF 296 kb) [file 10198_2019_1048_MOESM2_ESM.tif]

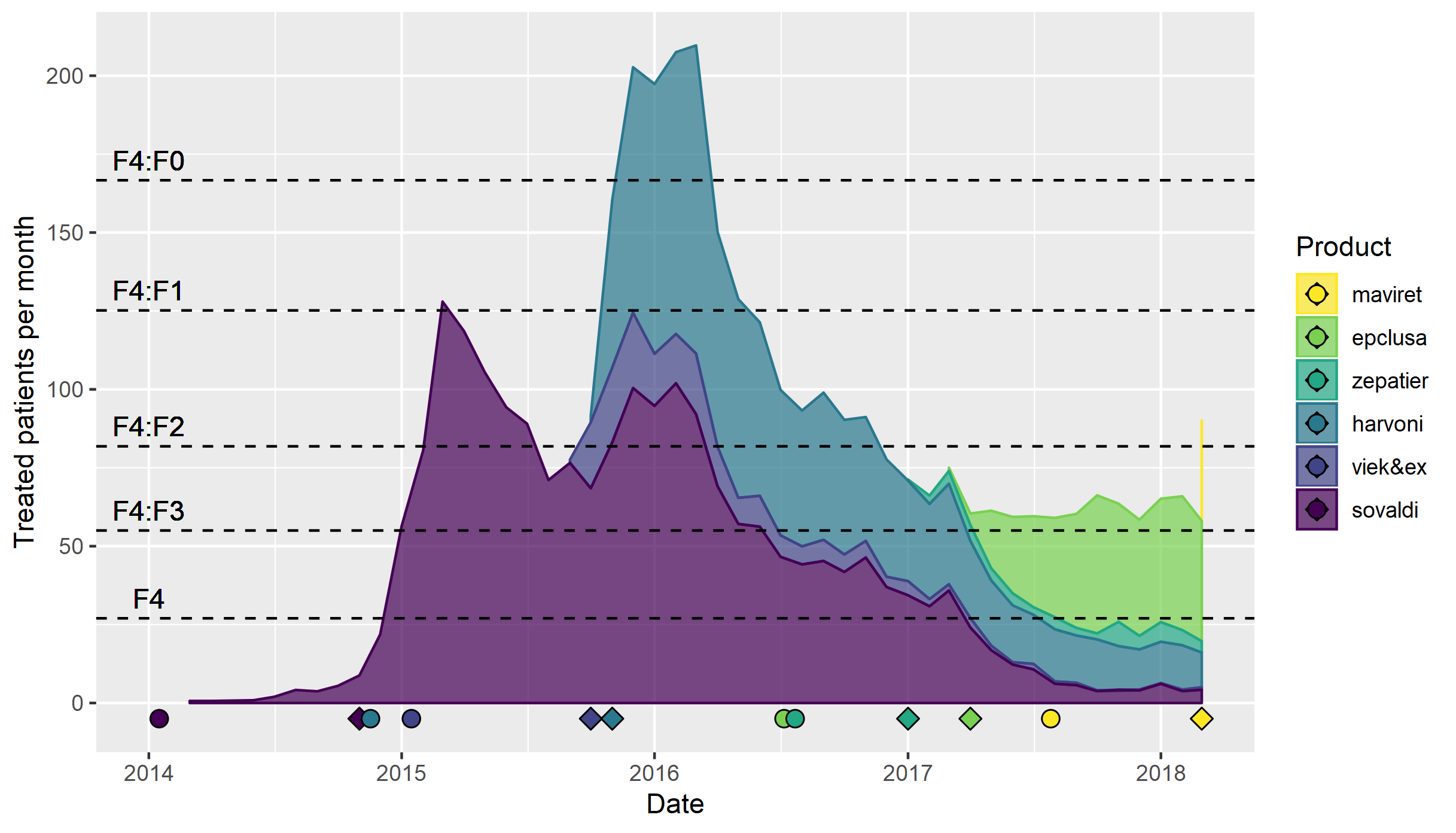

Supplement: Supplementary file 3 — Supplementary material 3 Base–case treatment costs, minimum population size of 2000 (TIFF 313 kb) [file 10198_2019_1048_MOESM3_ESM.tif]

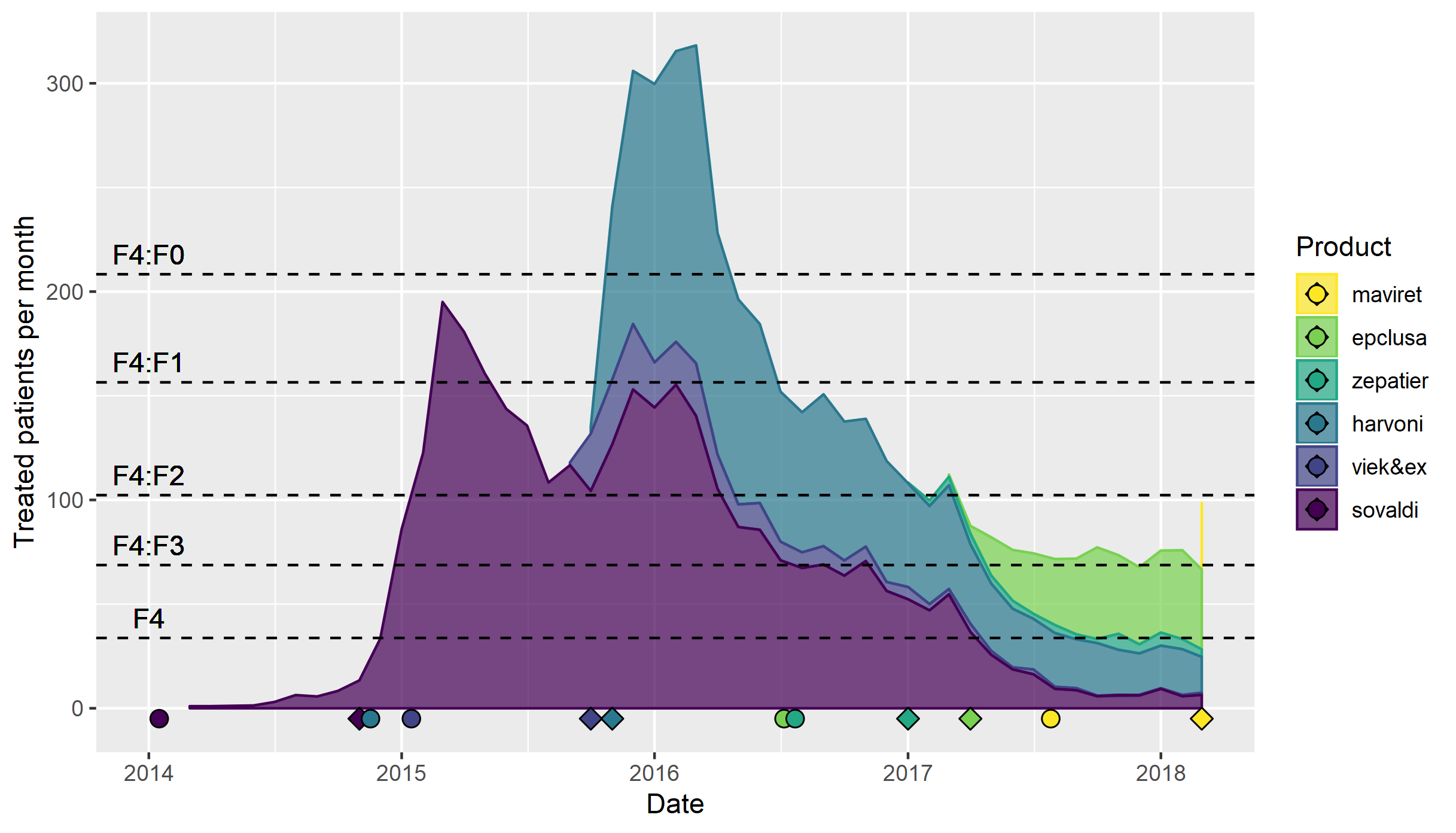

Supplement: Supplementary file 4 — Supplementary material 4 Minimum treatment costs, base–case population (TIFF 307 kb) [file 10198_2019_1048_MOESM4_ESM.tif]

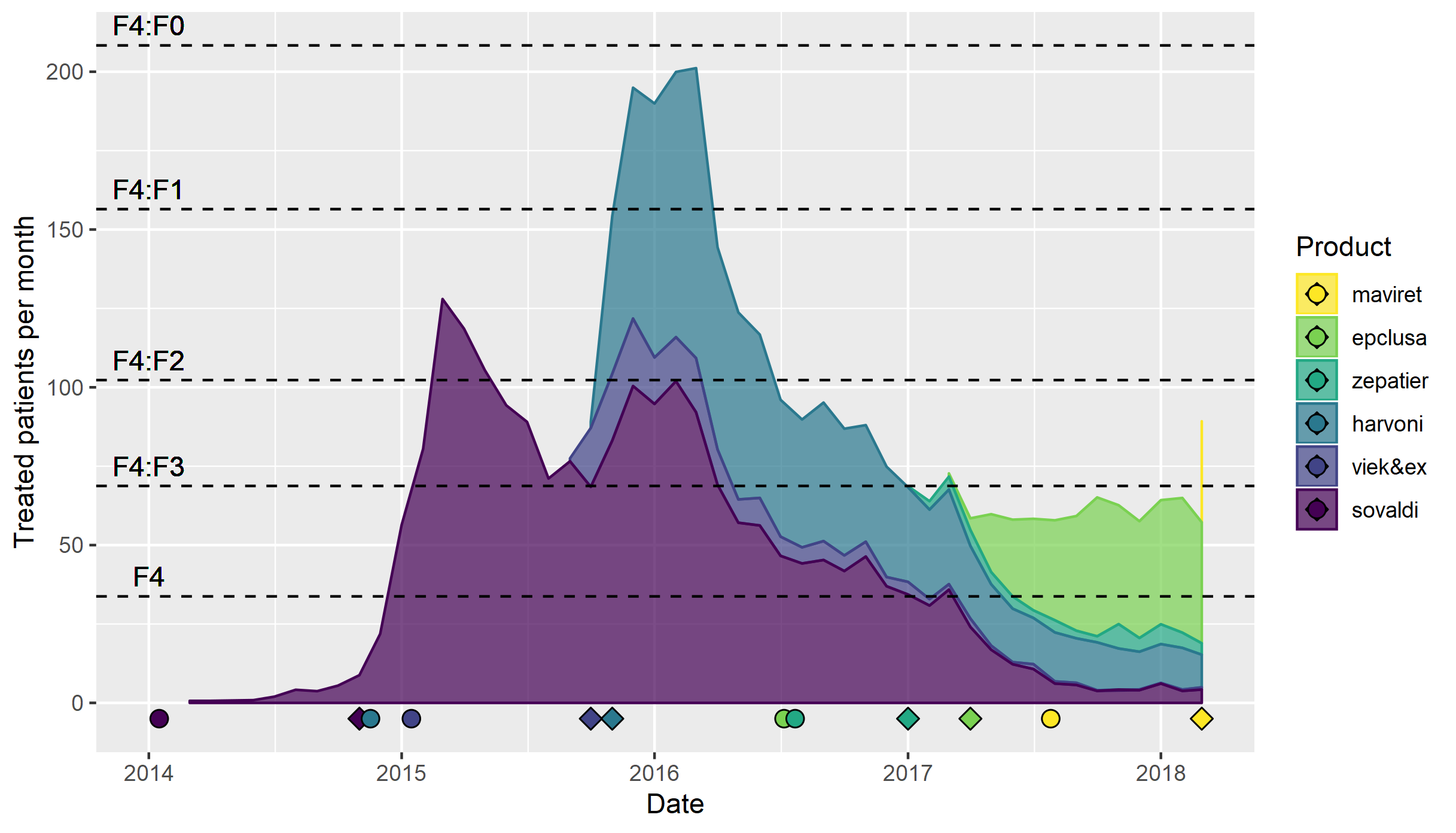

Supplement: Supplementary file 5 — Supplementary material 5 Scenario A treatment costs, base–case population (TIFF 310 kb) [file 10198_2019_1048_MOESM5_ESM.tif]

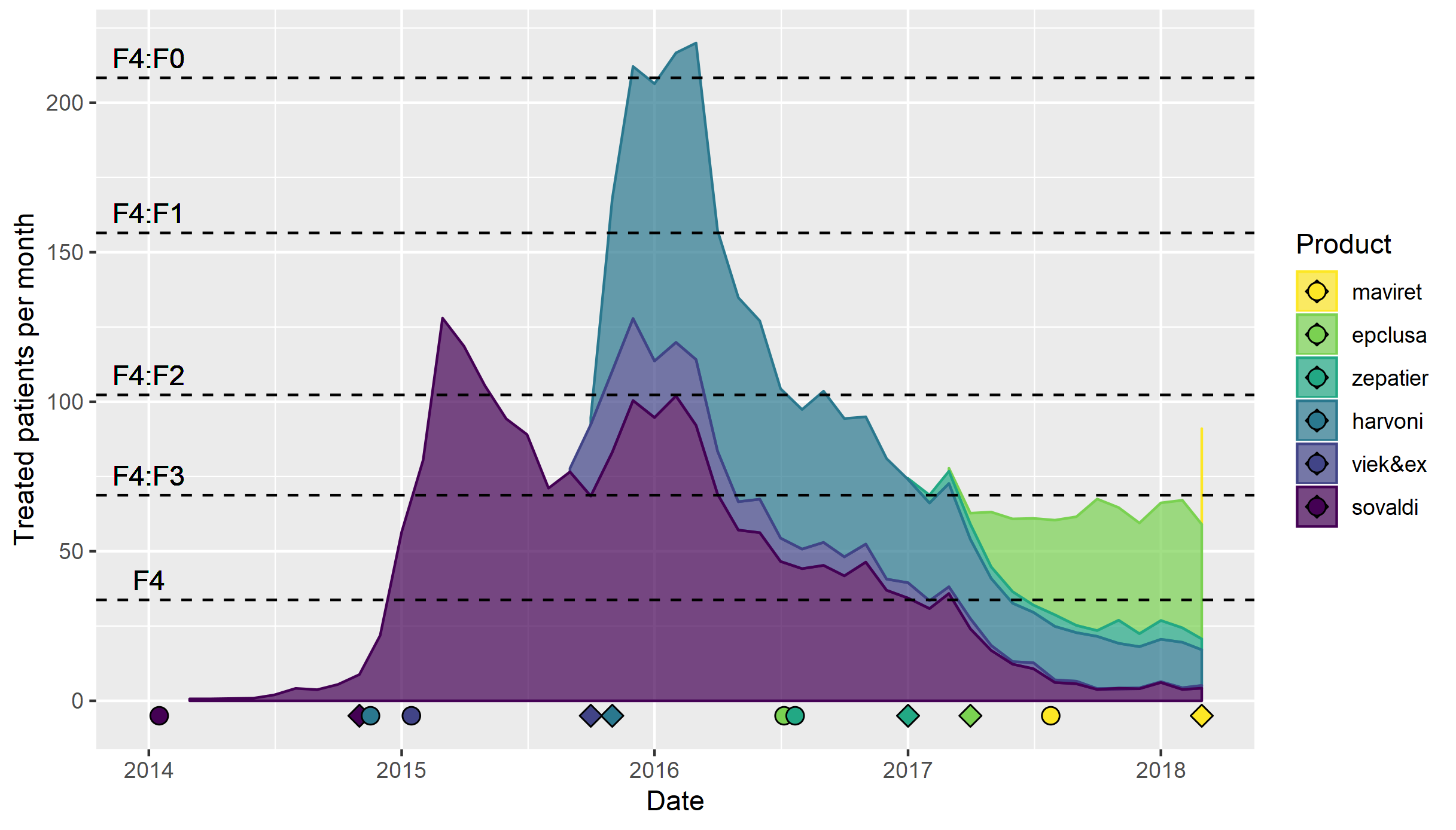

Supplement: Supplementary file 6 — Supplementary material 6 Scenario C treatment costs, base–case population (TIFF 309 kb) [file 10198_2019_1048_MOESM6_ESM.tif]
