# Supplementary material for: Accuracy of budget impact estimations and impact on patient access: a hepatitis C case study
Source: Eur J Health Econ. 2019 Apr 5;20(6):857–67. doi: 10.1007/s10198-019-01048-z (PMC6652171; doi:10.1007/s10198-019-01048-z)
Supplement: Supplementary file 7 — Supplementary material 7 (DOCX 14 kb) [file 10198_2019_1048_MOESM7_ESM.docx]

**Supplemental Table 1** Overview of extended treatment durations for specific subpopulations according to various EASL guidelines

| **Product** | **Subpopulation** | **Treatment duration (weeks)** |
| --- | --- | --- |
| **EASL 2014** | | |
| Sovaldi | GT1, GT4, IFN intolerant or ineligible | 24 vs 12. |
| Sovaldi + Daklinza | GT1, Tx experienced | 24 vs 12. |
| Sovaldi | GT2, F4, especially in Tx experienced | 16 – 20 vs 12. |
| Sovaldi | GT3, IFN-free | 24 vs 12. |
| Sovaldi + Daklinza | GT3, GT4, IFN-free in Tx experienced | 24 vs 12. |
| **EASL 2015** | | |
| Sovaldi | GT3, IFN-free, F0-F3 | 24 vs 12. |
| Harvoni | GT1, F0-F3 | 8-12 vs 12 |
| Sovaldi | GT2, IFN-free, F4 | 16 – 20 vs 12. |
| Viekirax + Exviera | GT1a, F4 | 24 vs 12 |
| Harvoni | GT1, GT4, F4, without RBV or with RBV if negative predictors of response | 24 vs 12 |
| Sovaldi + Olysio | GT1, GT4, F4, RBV-free | 24 vs 12 |
| Sovaldi + Daklinza | GT1 and GT4 RBV-free or GT3 with RBV, F4, | 24 vs 12 |
| **EASL 2016** | | |
| Harvoni | GT1, Tx naïve, F0-F3 | 8-12 vs 12 |
| Harvoni | GT1a, GT4, Tx experienced, F0-F3, RBV free | 24 vs 12 |
| Epclusa | GT3, Tx experienced, F0-F3, RBV free | 24 vs 12 |
| Viekirax + Exviera | GT1b, Tx naive, F0-F3 | 8 -12 vs 12 |
| Zepatier | GT1, HCV RNA >800,000 | 16 vs 12 |
| Zepatier | GT4, Tx experienced, HCV RNA >800,000 | 16 vs 12 |
| Sovaldi + Daklinza | GT1a, GT4, Tx experienced, RBV free | 24 vs 12 |
| Harvoni | GT1a, GT4, F4, Tx experienced, RBV-free | 24 vs 12 |
| Epclusa | GT3, F4, RBV-free | 24 vs 12 |
| Viekirax + Exviera | Gt1a, F4 | 24 vs 12 |
| Sovaldi + Daklinza | GT3, F4 | 24 vs 12 |
| **EASL 2018** | | |
| Maviret | GT3, F0-F3, Tx experienced | 12 vs 8 |
| Harvoni | GT1, F0-F3, Tx naive | 8-12 vs 12 |
| Zepatier | GT1b, F0-F2, Tx naive | 8 vs 12 |
| Viekirax + Exviera | GT1b, F0-F2, Tx naive | 8 vs 12 |
| Maviret | GT1, GT2, GT4, F4 | 12 vs 8 |
| Maviret | GT3, Tx naive, F4 | 12 vs 8 |
| Maviret | GT3, Tx experienced, F4 | 16 vs 8 |
