# Supplementary material for: Accuracy of budget impact estimations and impact on patient access: a hepatitis C case study
Source: Eur J Health Econ. 2019 Apr 5;20(6):857–67. doi: 10.1007/s10198-019-01048-z (PMC6652171; doi:10.1007/s10198-019-01048-z)
Supplement: Supplementary file 8 — Supplementary material 8 (DOCX 12 kb) [file 10198_2019_1048_MOESM8_ESM.docx]

**Supplemental Table 2** The Drug Information System of the National Health Care Institute data on Direct-Acting Antivirals in the Netherlands [33]. Numbers denote number of users (DDDs)

| **Product** | **2013** | **2014** | **2015** | **2016** | **2017** |
| --- | --- | --- | --- | --- | --- |
| Sovaldi | 0 | 77 (3826) | 1359 (128,560) | 1102 (90,269) | 292 (22,895) |
| Exviera | 0 | 0 | 84 (5584) | 149 (11,397) | 23 (1598) |
| Harvoni | 0 | 0 | 323 (18,813) | 1397 (104,540) | 524 (35,338) |
| Viekirax | 0 | 0 | 102 (6899) | 176 (13,240) | 28 (1906) |
| Zepatier | 0 | 0 | 0 | 0 | 61 (4532) |
| Epclusa | 0 | 0 | 0 | 0 | 274 (20,634) |
